# Chronic lymphedema reversal following arteriovenous fistula takedown: A case report

**DOI:** 10.1016/j.ijscr.2023.108519

**Published:** 2023-07-23

**Authors:** Lokesh S Jaiswal, Bijay Sah, Aakash Neupane, Nakul Regmi, Diwakar Koirala

**Affiliations:** B.P. Koirala Institute of Health Sciences, Nepal

**Keywords:** Arteriovenous fistula, Fistula takedown surgery, Lymphedema, Thrombosis

## Abstract

**Introduction and importance:**

Lymphedema is a very rare complication of Arteriovenous Fistula. The commonly encountered complications following the arteriovenous fistula are thrombosis, stenosis, congestive heart failure, ischemic neuropathy, steal syndrome, aneurysm and infection. Hence, presence of Lymphedema is a rarity that must be managed vigilantly. The incidence of lymphedema following AV fistula is very rare. Presently there is lack of studies evaluating the outcome of fistula take down. The standard care for lymphedema is complex decongestive physiotherapy in most of other causes bur Fistula Take down also helps in reducing the swelling in our case.

**Case presentation:**

Our case is of 53 years female presented to the surgical OPD with left upper limb swelling 5 months back which was non-pitting in nature. She was a known case of Acute kidney injury with no history of other comorbidities. The swelling started about 1 year ago involving the upper parts of the left arm which was intermittent and relieved spontaneously. She has a history of brachiocephalic fistula insertion for hemodialysis access 4 years ago with diagnosis of Acute Kidney Injury. However, the fistula was never used because of patient recovering from medical management. Investigations performed were doppler and other routine tests.

**Clinical discussion:**

The fistula was patent on examination confirmed by venous hum on auscultation. Fistula takedown surgery was planned after ruling out thrombosis and stenosis using doppler. Other alternatives were not considered because of lack of use of fistula. The swelling started to improve postoperatively and the patient was discharged.

**Conclusion:**

Our Case report highlights the fact that the rare complication like lymphedema could occur after the arteriovenous fistula which could be managed by fistula take down surgery if the fistula is no longer in use. Though very rare lymphedema should be kept in differential for complication which can be diagnosed by examination and ruling out other causes.

## Highlights

• Lymphedema following Arteriovenous Fistula
• Lymphedema is a very rare complication of Arteriovenous fistula.
• Suggested hypothesis is venous backflow causing fluid leakage.
• Lymphedema cured by Fistula Take down surgery.

## 1 Introduction

According to several studies, the development and complications of vascular access account for roughly 30 % of hospitalizations [1]. The conditions for greater blood flow through the venous system are created by AVF construction. Systemic venous pressure is approximately 20 mmHg before surgery, but it rises to between 60- and 120-mmHg within minutes of the procedure. Therefore, it's crucial to avoid the chance of obstructing blood flow when constructing the anastomosis. Making an acute angle, rotating the veins longitudinally, or altering their anatomical position is all prohibited. The likelihood of turbulence and endothelium damage is decreased when these requirements are met, which lowers the risk of stenosis. Surgical complications can be defined as complications during the intervention due to discrepancies between the lumen diameters of the arteries and veins, narrowing of the anastomosis, damaged intima-media, or interposition of the adventitia and the remaining collateral [2]. Immediately after surgery, hemorrhage, low venous flow or hematoma may occur. At a later stage, there may be complications, such as infections, the development of an aneurysm and/or false aneurysm, fistula vein stenosis, congestive heart failure, steal syndrome, ischemic neuropathy and thrombosis. Creation and maintenance of vascular access cannot exclude the occurrence of lymphedema [3]. Secondary lymphedema mainly occurs by impairment or obstruction of the lymphatic system due to disease or iatrogenic procedures.

## 2 Case report

### 2.1 Case history

A 53 years female presented to the surgical Out Patient clinic with left upper limb swelling 5 months back which was non-pitting. The swelling started about 1 year ago involving the upper parts of the left arm which was intermittent and relieved spontaneously. However, 5 months before presentation the swelling was persistent and not reliving even on elevating the limb above the level of the head. She had undergone a brachiocephalic fistula insertion for hemodialysis access 4 years ago. She had been diagnosed with Acute Kidney Injury with an abnormally high level of serum creatinine for which emergency dialysis was performed from the femoral vein, and fistula insertion was planned in anticipation of indications for repeated dialysis. However, the fistula was never used for hemodialysis as the patient improved on medical management only and repeated Renal Function tests were within normal limits during follow-up. She has no history of Hypertension, Diabetes Mellitus.

### 2.2 On examination

**Fig. 1:**
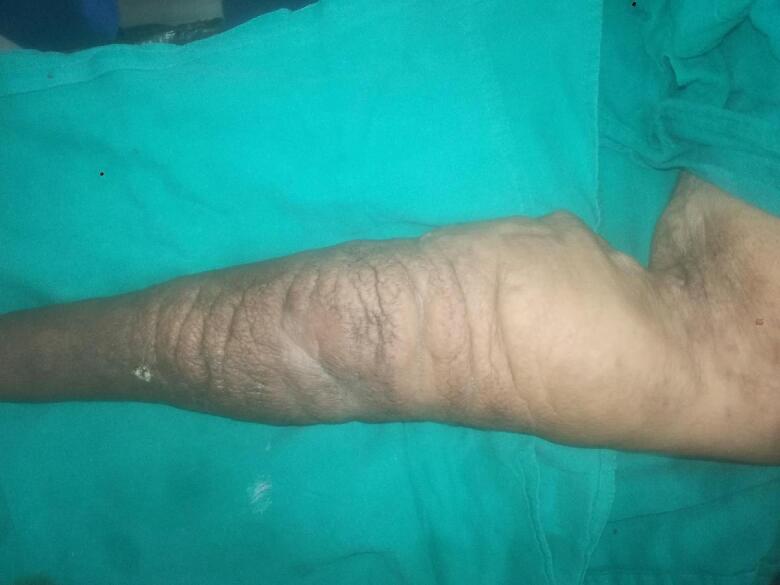
Pre-Operative Image

The left upper limb was enlarged from the Mid-upper arm to the fingertips. It was coarse on inspection and non-pitting on palpation. Fistula was patent on palpation and on auscultation, venous hum could be appreciated (Fig. 1).

### 2.3 On investigations

**Fig. 2:**
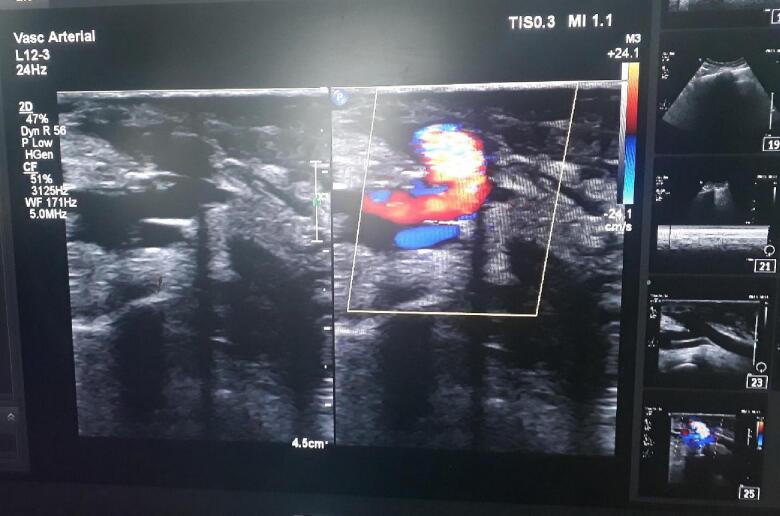
Doppler Image

**Fig. 3:**
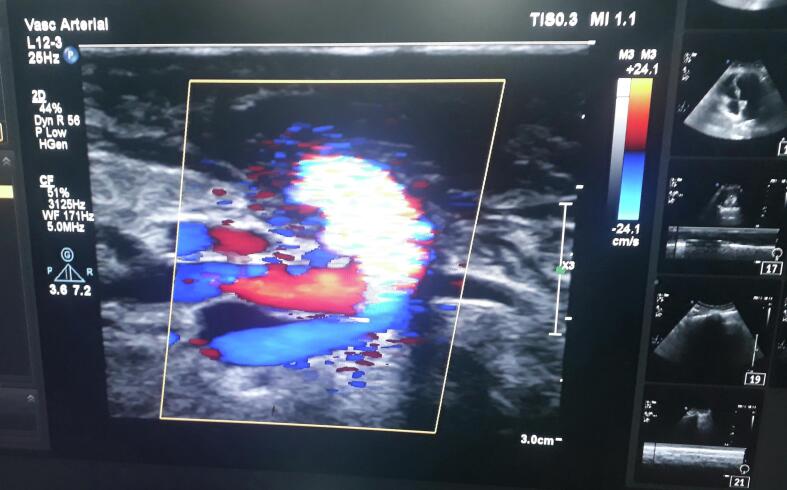
Doppler Image

Renal function test was normal, coagulation profile was normal, Hematology report showed Hb:8 g/dL, PCV:25.4 %, platelets: 1,45,000 cells/mm. ECG and Urine RE/ME were normal. Doppler ultrasonography was planned intraoperatively to rule out thrombosis and fistulography wasn't done under the clinician's hunch. Fig. 2, Fig. 3 show Doppler ultrasonography done intraoperatively.

**Fig. 4:**
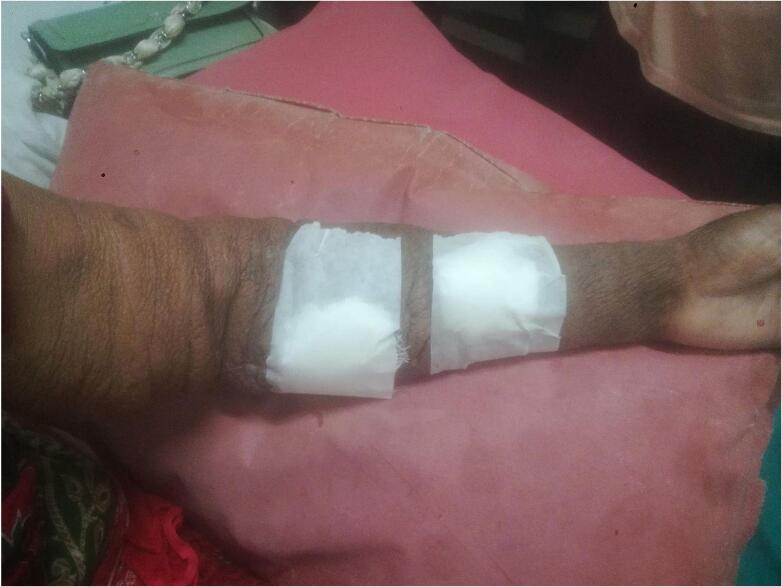
Post-Operative Image

As is evident from the picture the vein is dilated which rules out the possibility of thrombosis or stenosis. She was kept in the ward for observation during which furosemide was given to which the swelling responded but wasn't completely resolved so, Fistula takedown surgery was planned. Before the surgery, there was the presence of cyanosis on the fingernails, which resolved after the surgery was completed. Also, the swelling started to improve significantly and now it has been resolved completely. Postoperatively, she was prescribed some injectable antibiotics and analgesics along with transfusion of one-pint PCV. Fig. 4 shows the limb after the fistula takedown surgery. Antibiotics were used despite the surgery being clean one as a prophylaxis measure to prevent any secondary infections. This was done considering the socioeconomic background and feasibility of patient to come to hospital in the case of any abnormality observed.

**Fig. 5:**
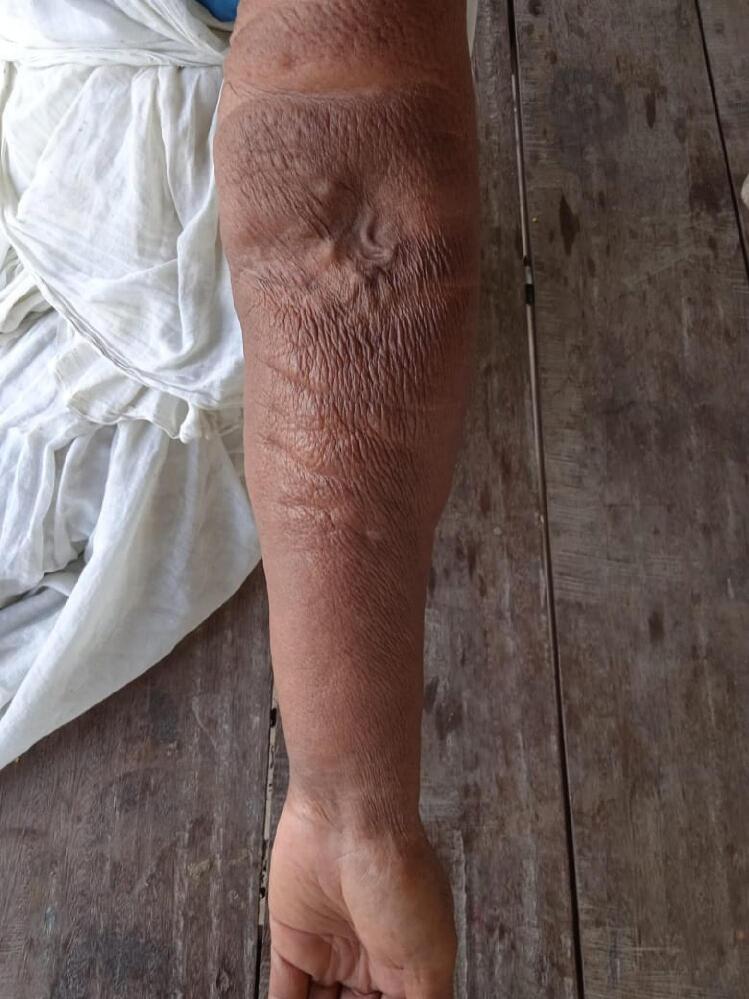
Post-Operative Image after 1 Year of Fistula Take down

Fig. 5 shows the same hand one year after fistula take down surgery.

## 3 Discussion

A high proportion of Arteriovenous Fistula fails to mature for use in dialysis [4,5]. The commonly encountered complications following the arteriovenous fistula are thrombosis, stenosis, congestive heart failure, ischemic neuropathy, steal syndrome, aneurysm and infection [3]. Sometimes limb hypertrophy may be present due to an increase in blood flow to the affected limb. However, very few cases have been reported where lymphedema was a chronic complication of arteriovenous fistula formation. Patients receiving Sirolimus after renal transplant for chronic kidney disease had higher risk of developing upper limb lymphedema [6,7]. In our case we propose lymphedema as a complication following long standing Arteriovenous Fistula. However, there are very less studies of lymphedema following arteriovenous fistula. Kono et al. described the presence of micro-arteriovenous fistula in patient with lower limb edema and suggested the hypothesis that venous blood pressure due to fistula causes leakage of plasma components and chronic leakage leads to increased lymph production and fibrosis around the lymph duct [14]. Majority of the cases that have been reported have involved lower limbs predominantly. In this case, the swelling was probably due to lymphatic obstruction due to venous hypertension as mentioned by Akoh et al. [8]

Alev Ôzçete et al. reported a case of a 57-year female with lymphedema in the left upper limb with a history of bilateral mastectomy with left axillary dissection and end-stage renal failure had arteriovenous fistula in the left arm for dialysis access [9]. However, she was treated with kinesio taping because the arteriovenous fistula needed to be intact for dialysis in contrary to our case.

Many cases have been reported where lymphedema has occurred in patient but the reversible causes were identified and lymphedema resolved. Pinho et al. described a case of an 87-year-old female patient with grade 3 lymphoedema of the upper right extremity which had developed over the course of two years and investigation identified a thyroid tumour, which compressed the mediastinal structures at the right superior thoracic outlet, causing venous congestion, oedema, and lymphoedema. The patient underwent thyroidectomy which markedly improved the lymphatic oedema.^16^ Similarly, McFarlane et al. described a case of an 84-year-old woman presented with a 3-year history of unilateral swelling of the right upper limb with no constitutional symptoms and no evidence of lymphadenopathy or systemic disease with blood tests, carcinoembryonic antigen test, computed tomography scans, and venous Doppler ultrasound all found normal. The diagnosis was primary upper limb lymphedema.^15^

Li Min et al. described a case of lymphedema of upper extremity induced by prolonged sun exposure 11 years after breast cancer surgery. She was started on eight days, 400 U (B.I.D.) penicillin and elastic sleeve twice daily for 30 min with sleeve pressure less than 40mmhg. She got comprehensive recovery within 15 days of admission and there was no complaint of discomfort after the follow up visits.^17^

Lower limb lymphedema was found to be more common than upper limb. However, similar to upper limb lymphedema, most of the swelling resolved after removal of the insult. Thompson et al. reported a case of 62 years lady with a right lower limb swelling and painful varicosities with an arteriovenous graft for hemodialysis 5 years back. All the complications were ruled out and it was confirmed to be a case of lymphedema [10]. The case was managed conservatively because of decreased eGFR with the requirement of dialysis in the future. However, in our case the requirement of dialysis was deemed unnecessary hence, the approach was surgical.

Jin et al. reported a case of 42 years old Asian man with a history of diabetic ketoacidosis and Acute Renal Failure admitted to the hospital and underwent emergency hemodialysis [12]. The patient developed lower limb swelling. On color Doppler, an arteriovenous fistula was detected between the right superficial femoral artery and the right femoral vein. The fistula was repaired through vascular surgery using running non-absorbable sutures and the patient was discharged after 8 days.

Kalender et al. reported a case of 52 years old white male patient with complaints of palpitations, pain and swollen right leg [11]. He had suffered from penetrating injury from a gunshot in the right thigh 5 years ago. Fistula was detected 3 years ago but didn't receive any treatment because of his choice. His symptoms exaggerated in the last 2 months. Angiography revealed an arteriovenous fistula between the common femoral artery and common femoral vein which was repaired and patients' symptoms improved significantly.

## 4 Conclusion

This case demonstrates the possibility of Lymphedema as a late complication of Arteriovenous Fistula though very rare. Also, all the causes like thrombosis, Stenosis and steal phenomenon must be ruled out before the diagnosis of lymphedema. The management of lymphedema also depends on the indication of dialysis in the patient. If the patient has end-stage renal disease, then conservative management must be preferred as dialysis would be needed. However, as evident from our case if dialysis is not indicated then the fistula takedown procedure can be done.

## Informed consent

Informed consent was taken from the patient explaining all the process and procedures.

## Additional information

Author declares no conflict of interest, sources of funding, and has been approved from Institutional Review Committee.

## Methodology

The work has been reported in line with the SCARE criteria [13].

## Ethical approval

Ethics approval not required for case reports as per BPKIHS Institutional Review Committee. Because it doesn't involve any additional intervention except the treatment of the patient.

## Consent

Written informed consent was obtained from the patient for publication of this case report and accompanying images. A copy of the written consent is available for review by the Editor-in-Chief of this journal on request.

## Funding

The Study had no funding involved.

## Guarantor

Dr. Lokesh Shekhar Jaiswal.

Dr. Bijay Sah.

Dr. Aakash Neupane.

Dr. Nakul Regmi.

Mr. Diwakar Koirala.

## CRediT authorship contribution statement

**Lokesh S Jaiswal:** Conceptualization, Data curation, Investigation, Methodology, Validation, Supervision. **Bijay Sah:** Conceptualization, Validation, Data curation, Investigation, Methodology, Validation, Supervision. **Aakash Neupane:** Investigation, Methodology, Validation, Data curation, Methodology, Visualization, Writing – original draft, Writing – review & editing. **Nakul Regmi:** Writing – original draft, Writing – review & editing, Data curation, Investigation, Methodology, Visualization, Supervision. **Diwakar Koirala:** Writing – original draft, Writing – review & editing, Data curation, Investigation, Methodology, Visualization.

## Declaration of competing interest

The author declares no conflict of interest.

## Data Availability

No Data sheets were used to analyse the data as it is a case report.
